# Introducing new plan evaluation indices for prostate dose painting IMRT plans based on apparent diffusion coefficient images

**DOI:** 10.1186/s13014-022-02163-7

**Published:** 2022-11-23

**Authors:** Saman Moradi, Bijan Hashemi, Mohsen Bakhshandeh, Amin Banaei, Bahram Mofid

**Affiliations:** 1 Department of Medical Physics, Faculty of Medical Sciences, Tarbiat Modares University, Tehran, 1411713116 Iran; 2 Department of Radiology Technology, Faculty of Allied Medical Sciences, Shahid Beheshti University of Medical Sciences, Tehran, 1985717443 Iran; 3 Department of Radiation Oncology, Faculty of Medicine, Shahid Beheshti University of Medical Sciences, Tehran, 1985717443 Iran

**Keywords:** Prostate cancer, Intensity-modulated radiotherapy, Radiobiology, Anatomical parameters, Algorithmic approach

## Abstract

**Background:**

Dose painting planning would be more complicated due to different levels of prescribed doses and more complex evaluation with conventional plan quality indices considering uniform dose prescription. Therefore, we tried to introduce new indices for evaluating the dose distribution conformity and homogeneity of treatment volumes based on the tumoral cell density and relative volumes of each lesion in prostate IMRT.

**Methods:**

CT and MRI scans of 20 male patients having local prostate cancer were used for IMRT DP planning. Apparent diffusion coefficient (ADC) images were imported to a MATLAB program to identify lesion regions based on ADC values automatically. Regions with ADC values lower than 750 mm^2^/s and regions with ADC values higher than 750 and less than 1500 mm^2^/s were considered CTV_70Gy_ (clinical tumor volume with 70 Gy prescribed dose), and CTV_60Gy_, respectively. Other regions of the prostate were considered as CTV_53Gy_. New plan evaluation indices based on evaluating the homogeneity (IOE(H)), and conformity (IOE(C)) were introduced, considering the relative volume of each lesion and cellular density obtained from ADC images. These indices were compared with conventional homogeneity and conformity indices and IOEs without considering cellular density. Furthermore, tumor control probability (TCP) was calculated for each patient, and the relationship of the assessed indices were evaluated with TCP values.

**Results:**

IOE (H) and IOE (C) with considering cellular density had significantly lower values compared to conventional indices and IOEs without considering cellular density. (P < 0.05). TCP values had a stronger relationship with IOE(H) considering cell density (R^2^ = -0.415), and IOE(C) without considering cell density (R^2^ = 0.624).

**Conclusion:**

IOE plan evaluation indices proposed in this study can be used for evaluating prostate IMRT dose painting plans. We suggested to consider cell densities in the IOE(H) calculation formula and it’s appropriate to calculate IOE(C) without considering cell density values.

**Supplementary Information:**

The online version contains supplementary material available at 10.1186/s13014-022-02163-7.

## Background

Modulated radiotherapy techniques such as intensity modulated radiotherapy (IMRT) and volumetric modulated arc therapy (VMAT) are among the most useful techniques for prostate radiotherapy [1], to deliver the prescribed dose to the target tissue and spare organs at risk (OARs) from irradiation. Although VMAT generally has a smaller treatment time and slightly better OAR sparing [2, 3], however, IMRT has indicated similar outcomes to VMAT and also newer and more complicated radiotherapy techniques such as Tomotherapy [4–7].

One of the strategies to improve the efficiency of radiotherapy is increasing the prescribed dose in regions with higher cancer cell densities [8]. Higher doses ranging from 74 to 80 Gy has been reported to have improvements in the outcome of prostate cancer treatment [4–7, 9–14]. However, delivering these high doses is impossible using conventional radiotherapy without a significant probability of occurring severe radiation toxicities [14]. Modulated techniques (IMRT, VMAT, Tomotherapy) can reduce these toxicities by optimizing radiation conformation [15, 16].

The usual clinical protocol in prostate radiotherapy is to deliver a uniform dose to a defined planning target volume (PTV) [17]. Dose painting (DP) was introduced to increase the tumor local control rates by delivering higher dose levels to the regions with higher cellular densities or radioresistance tissues while sparing healthy tissue. It is designed to give additional doses to subvolumes with high radioresistance due to hypoxia or other reasons as quantified by functional imaging [18]. Higher dose levels in tumor nodules or dominant intraprostatic lesions can improve local control without increasing complication rates [4–7, 14, 15].

In dose painting, tumors and intra-tumoral lesions (with higher cell densities) are delineated based on multiparametric MRI, consisting of a T2-weighted (T2w), diffusion-weighted (DWI), apparent diffusion coefficient (ADC), and a dynamic contrast-enhanced (DCE) sequences [19, 20]. Other imaging modalities such as positron emission tomography (PET) with ^18^F compounds (like ^18^F-FDG, ^18^F-choline, ^11^C-choline and ^18^F-Fluoromisonidazole) are also available and their applications were reported for delineating intra tumor lesions [21–24]. As an alternative to manual contouring, automated methods for the prostate have been developed [25–29].

The application of DP radiation therapy is increased in recent years. However, the aspects of plan evaluation remain controversial now [30]. For example, conventional indices, such as the conformity index (CI) and the homogeneity index (HI), commonly used in routine clinical practice for plan evaluation, are formulated based on the paradigm of uniform dose prescription. Therefore, these indices need to be modified for DP planning. There are few studies that modified these indices for use in DP plan evaluation [31–33]. In a study by Park et al. [31], they introduced a new plan quality index, named “index of achievement (IOA)”. Their introduced index assesses how close the planned dose distribution is to the prescribed one considering the differences between the prescribed and the delivered dose for each voxel multiplied by the relative volume of the voxel in the target volume. Their index did not account for the importance of different lesions with different cellular densities or radio-resistance properties and also need to be calculated by a computer program. We think that the plan evaluation index in DP planning must be easy to calculate without a computer program and accounts importance of different lesions inside target volume. Therefore, in this study we tried to propose new dose painting plan evaluation indices with simpler calculation methods incorporating cellular density as an index of lesion importance obtained from MRI diffusion images for prostate cancer IMRT.

## Methods

This single-center retrospective study was performed in accordance with the national ethical guidelines and regulations. The national Ethics Committee has approved the methods of this study. MRI and CT images of patients were used in this study without any intervention in the diagnostic or treatment procedures. In addition, gathering the informed consent was waived because of the retrospective nature of the study.

Imaging data (CT and MRI scans) of 20 male patients having local prostate cancer who had no previous surgery, hormone therapy (AST or ADT), and prostate radiation therapy with at least one non-high risk intraprostatic lesion (IL) in stages of T1 to T3a, were used in this study. Patients’ ages ranged from 54 to 85 years, with a mean age of 69.4.

The CT (Matrix size: 512*512; Slice thickness: 3 to 5 mm), T2w-MRI (fast spin echo pulse sequence with TE: 80 ms and TR: 7800 ms), diffusion-weighted MRI (echo planar imaging with TE: 88 ms, and TR: 4600 ms) and apparent diffusion coefficient (fast spin echo pulse sequence with TE: 100 ms and TR: 3000 ms) images were taken using a Siemens 16-slice Emotion CT and a 1.5 Tesla Avanto MRI machine (Siemens Healthcare GmbH, Germany). The patients were placed in supine positions for both imaging procedures. Diffusion-weighted images (DWI) were gathered with three signals per image with a scattering-sensitive gradient in three orthogonal planes and b-values of 0, 250, 500, and 1000 s per square. DW-MRI images have a resolution of 1.64 × 1.64 × 3 mm and a FOV of 210 × 210 mm, a matrix size of 128 × 128 pixels, and a NEX (number of excitation) parameter equal to four. Apparent diffusion coefficient (ADC) maps (images) were automatically calculated from DW-MRI images. ADC is a measure of the magnitude of water molecules diffusion within tissue [34], and can show the cellular density in some tumors like prostate [35].

CT and MRI images were combined using a rigid registration algorithm in the treatment planning software (TPS) based on bone landmarks, gold markers implanted in the prostate and skin surfaces, and then verified by a specialized physician. The MRI and CT registration were used to contour the lesion volumes inside the patients’ prostate and radiation-sensitive organs. It also allows for more precise target volume delineation in prostate cancer patients [36].

An in-house MATLAB program was developed to automatically identify lesion regions on ADC images based on ADC values. The MATLAB code is available in the “Additional file 1” section. Two types of predominant lesions were considered in the prostate, one related to lesions with ADC values lower than 750 mm^2^/s (PTV-1), and the other was related to lesions with ADC values higher than 750 and less than 1500 mm^2^/s (PTV-2). The upper limit of ADC values in tumor tissues varies in different studies but usually was considered more than 1300 mm^2^/s [37]. In this study, the apparent diffusion coefficient threshold for distinguishing tumor tissue from normal prostate tissue was 1500 mm^2^/s. The highest measurable value of ADC in MATLAB software was 5000 mm^2^/s. The ADC images were imported to the MATLAB program, and the voxels in a specific range of ADC values were determined. The related voxels for determining areas must at least have a minimum number (400 voxels) located next to each other so that the software can identify those areas separately. Considering the relationship between Gleason score (GS) and ADC cut-off value based on a study by Pepe et al. [38], different target volumes were identified within the prostate. Each patient's output DICOM RS file was then transferred to the treatment planning system to contour these new structures (prostatic lesions) on the CT images. Contouring of other organs at risk (OARs) was performed by an experienced radiation oncologist in the treatment planning system.

**Fig. 1:**
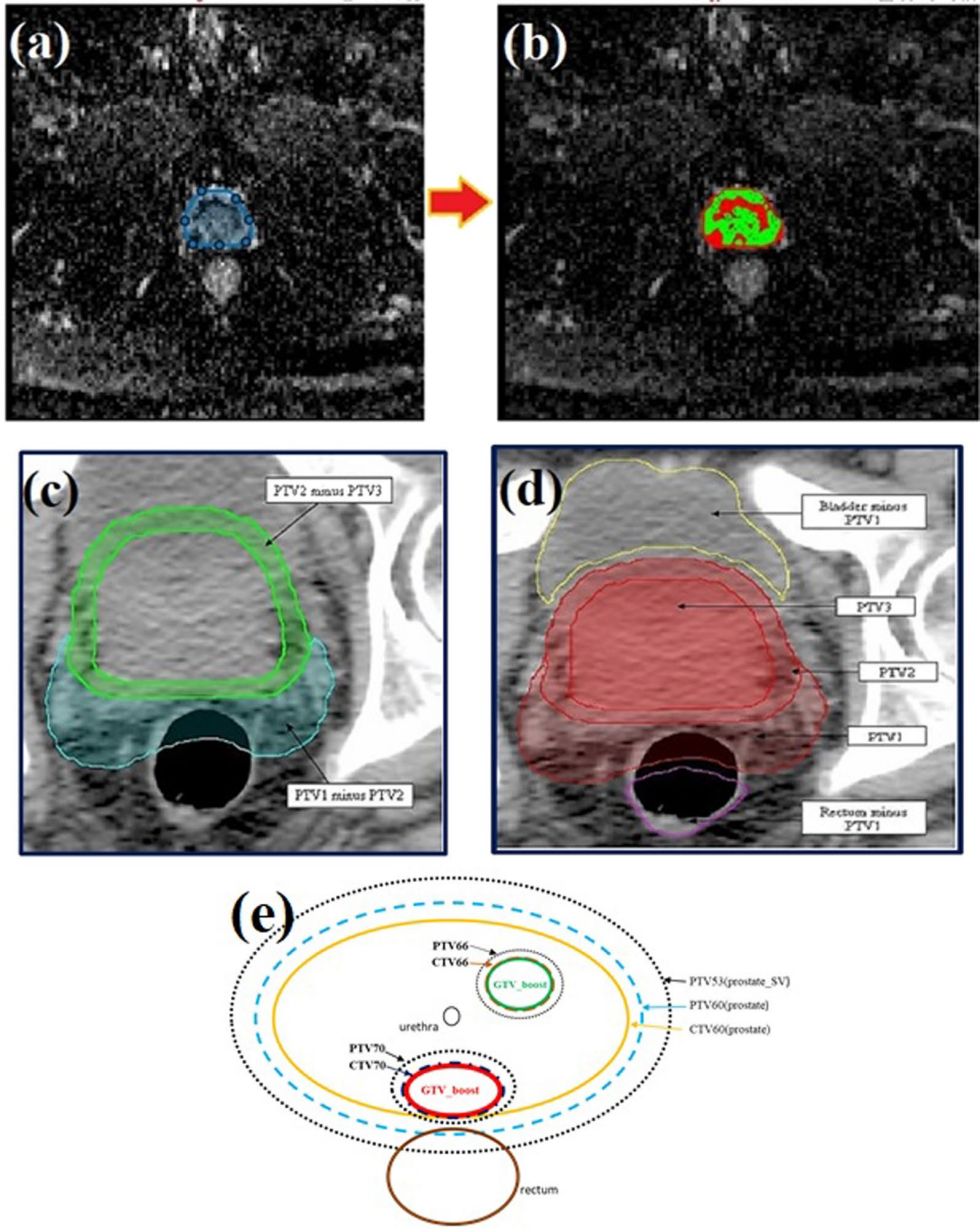
The procedure used to automatically contour the intraprostatic dose escalated DILs. **a** Delineating the whole prostate manually on ADC images. **b** Automatic contouring of different intraprostatic lesions based on ADC values. **c** Importing the contours from ADC images on registered CT images. **d** Determining DILS and margins to create different DILs with different dose levels on CT images. **e** Schematic of intraprostatic DILs and their PTVs with different dose levels

Prescription dose levels, except in dominant intraprostatic lesions (DILs), were taken from the Jereczek-Fossa et al. study [39]. The upper limit for the prescribed dose for DIL was considered 70 Gy in 27 fractions for high risk DILs. This hypofractionated dose escalation was used and evaluated in many studies [40–47]. According to a study by Onjukka et al. [48], this hypofractionated dose was equivalent to 86 Gy in 37 sessions (used in the study of Uzan et al. [49]. A prescribed dose for DILs with lower risks was considered 66 Gy. The clinical target volume of the base of the seminal vesicles was considered CTV_53Gy_. The planning target volume for this target (PTV_53Gy_) was formed by adding eight millimeter isotropic margins to CTV_53Gy_ in order to account for patient and equipment placement errors. The whole prostate volume (except the DILs) was considered CTV60; similarly, PTV_60Gy_ was formed by adding 5 mm margins to the CTV_60Gy_. The margin was reduced to zero in the posterior region, where the target volume overlaps with the rectum. Two millimeters margins were added to CTV_66Gy_ and CTV_70Gy_ to create PTV_66Gy_ and PTV_70Gy_ without extending beyond CTV_60Gy_ or overlapping with the rectum, bladder, and urethra due to the uncertainty in defining the DILs [49]. Furthermore, planning at risk volumes (PRVs) were created for high-risk organs, including the rectum, urethra, and bladder, with margins of two millimeters. The dose escalated DIL regions with the whole prostate were presented in Fig. 1. Furthermore, the procedure used to automatically contour the intraprostatic DILs is illustrated in Figs. 1-a and 1-b.

The IMRT plans were designed with Eclipse software (version 11, Varian Corporation, USA) for each patient. IMRT plan with nine coplanar fields in gantry angles of 0, 30, 60, 105, 140, 220, 260, 300, and 330 was designed to irradiate PTVs with prescribed doses. All the plans were interactively optimized based on our institutional planning protocol derived from a previous study by Pollak et al. [50]. The optimization algorithm was Dose Volume Optimizer (DVO) which is enclosed in Eclipse treatment planning software and clinically approved by previous studies [51]. The planning optimization objectives are presented in Table 1. An experienced physicist evaluated all the treatment plans to ensure compliance with reported dose constraints [52].

**Table 1:** The planning optimization objectives used for prostate dose painting IMRT

Structures	Objectives
Bladder	V_40.8 Gy_ < 50%
	V_48.6 Gy_ < 25%
	V_60Gy_ < 5%
	Max dose < 65 Gy
Rectum	V_40.8 Gy_ < 50%
	V_48.6 Gy_ < 35%
	Max dose < 65 Gy
	V_60Gy_ < 3%
Femoral heads	Max dose < 40 Gy
Bowel	V_50Gy_ < 17 cc
	Max dose < 60 Gy
PTVs	V_98%_ > 98%
	V_105%_ < 2%

^*^V_xGy_ represents the percentage of the structure volume received at least x Gy

^*^V_x%_ represents the percentage of the structure volume received at least x% of the prescribed dose

After treatment planning optimization, final dose calculations were performed by the anisotropic analytical algorithm (AAA) in the Eclipse software. The calculation accuracy of this algorithm was previously approved in several studies [53–55]. Dose volume histograms (DVHs) of the CTVs for each patient were entered in BioSuite software [56]. The tumor control probability (TCP) values were calculated using the Poisson model [57], based on radiobiological model parameters proposed by Deb and Fielding [58].

We introduced two indices of effectiveness (IOE) for evaluating IMRT dose painting plan dose distribution. One IOE can evaluate the conformity of CTVs, IOE(C), and another IOE can assess the overall dose distribution homogeneity of target volumes, IOE(H). The previous equation proposed by Park et al. [31] has relative volume coefficients, and these coefficients were included in our IOE equations accounting for the effect of each target (DIL) on the overall value of IOE. Furthermore, cell density values obtained from ADC maps were used in the IOE equations. The cell density is a measure of the clonogenicity level for each of the tumor volumes. The equations of IOE(H) and IOE(C) are as follows:

1 $$IOE\left(H\right)=\left({HI}_{1}\times \frac{{CD}_{1}}{{CD}_{p}}\times \frac{{V}_{1}}{{V}_{T}}\right)+\left({HI}_{2}\times \frac{{CD}_{2}}{{CD}_{P}}\times \frac{{V}_{2}}{{V}_{T}}\right)+\left({HI}_{3}\times \frac{{CD}_{3}}{{CD}_{P}}\times \frac{{V}_{3}}{{V}_{T}}\right)$$

2 $$IOE\left(C\right)=\left({CI}_{1}\times \frac{{CD}_{1}}{{CD}_{p}}\times \frac{{V}_{1}}{{V}_{T}}\right)+\left({CI}_{2}\times \frac{{CD}_{2}}{{CD}_{P}}\times \frac{{V}_{2}}{{V}_{T}}\right)+\left({CI}_{3}\times \frac{{CD}_{3}}{{CD}_{P}}\times \frac{{V}_{3}}{{V}_{T}}\right)$$

HI_1_, HI_2_, and HI_3_ are the homogeneity indices for CTV_70Gy_, CTV_66Gy_, and CTV_60Gy_ respectively. Similarly, the CI_1_, CI_2_, and CI_3_ are the conformity indices for CTV_70Gy_, CTV_66Gy_, and CTV_60Gy_ respectively. V_1_, V_2_, and V_3_ are the volumes, and CD_1_, CD_2_, and CD_3_ are the cell density of these CTVs. In addition, V_T_ and CD_P_ are the total volume and mean cell density value of the whole CTV (sum of all CTVs).

Furthermore, the IOE(C) and IOE(H) were calculated without considering the cell density, and they were compared with IOE indices considering cell density (our proposed indices) and also the mean of the conventional HI and CI values. Equations related to IOE indices without considering cell densities and the mean of conventional indices are presented in Eqs. 3–6.

3 $$IOE\left(H\right)=\left({HI}_{1}\times \frac{{V}_{1}}{{V}_{T}}\right)+\left({HI}_{2}\times \frac{{V}_{2}}{{V}_{T}}\right)+\left({HI}_{3}\times \frac{{V}_{3}}{{V}_{T}}\right)$$

4 $$IOE\left(C\right)=\left({CI}_{1}\times \frac{{V}_{1}}{{V}_{T}}\right)+\left({CI}_{2}\times \frac{{V}_{2}}{{V}_{T}}\right)+\left({CI}_{3}\times \frac{{V}_{3}}{{V}_{T}}\right)$$

5 $${HI}_{mean}=\left(\frac{{HI}_{1}+{HI}_{2}+{HI}_{3}}{3}\right)$$

6 $${CI}_{mean}=\left(\frac{{CI}_{1}+{CI}_{2}+{CI}_{3}}{3}\right)$$

### Statistical analysis

Kolmogorov–Smirnov (K–S) test was used to evaluate the variables' normality distribution. The results showed that the distributions of the assessed parameters for 20 patients studied in this study were not normal. Therefore, the Wilcoxon statistical analysis was used to evaluate the differences in the relevant indices with considering cell density, without considering cell density and the mean of conventional indices. The significance level in these tests was considered equal to 5%, and the P-values less than 0.05 were considered significant differences. Spearman test was also used to investigate the relationship between IOE indices and radiological parameter (TCP of different targets) values. All of the statistical tests were performed in the SPSS software package Version 22 (SPSS Inc., Chicago, IL, USA).

## Results

The mean and standard deviation of HI values for clinical target volumes including CTV_70Gy_, CTV_66Gy_, CTV_60Gy_, and CTV_53Gy_ were 0.086 ± 0.011, 0.078 ± 0.005, 0.151 ± 0.016, 0.105 ± 0.007, respectively. HI values for CTV_70Gy_ and CTV_66Gy_ were lower than the HI values of CTV_60Gy_, indicating a more homogeneous dose distribution in CTV_70Gy_ and CTV_66Gy_ volumes. Mean and standard deviation values of conformity index (CI) for these CTVs were 0.992 ± 0.005, 0.998 ± 0.004, 0.992 ± 0.004, 0.994 ± 0.004, respectively.

**Fig. 2:**
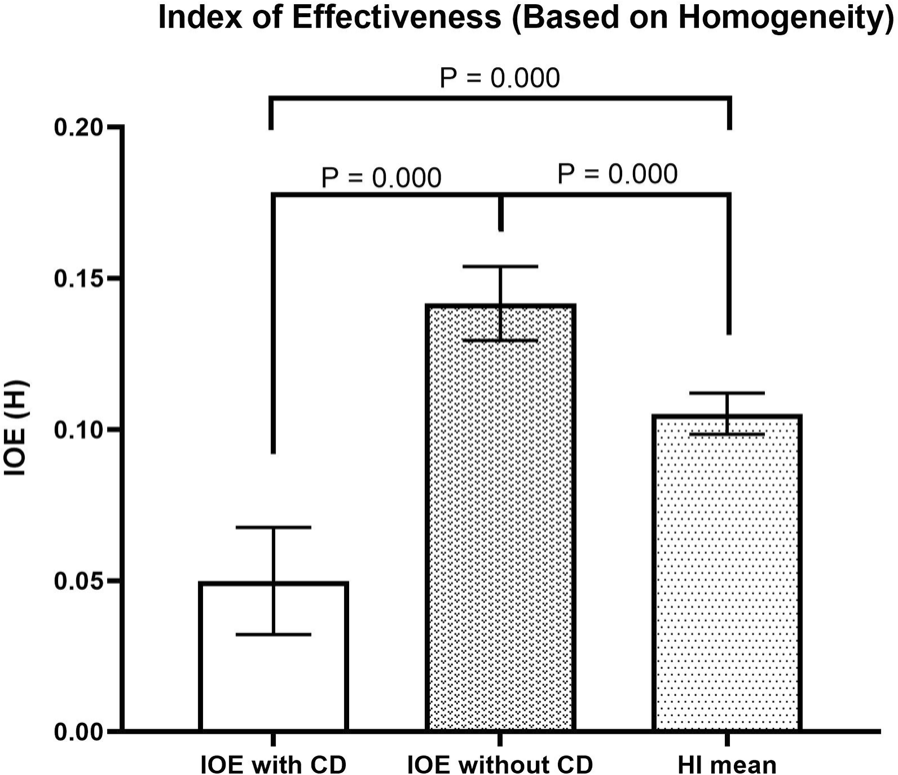
Mean and standard deviation (error bars) values of the IOE (H) with and without considering cell density coefficients and also the average of conventional HI values. *P-values lower than 0.05 represents the statistically significant differences

Similar to conventional HI and CI values, IOE (H) values closer to zero indicate the greater effectiveness of the treatment plan. In contrast, IOE (C) values closer to one indicate greater dose painting treatment plan conformity. Figure 2 shows the mean and standard deviation values of the IOE (H) with and without considering cell density coefficients and also the average of conventional HI values. The IOE (H) values with considering cell density had lower values compared to this index without considering cell density. In addition, IOE (C) without considering cell density had higher values compared to this index with considering cell density.

Mean and standard deviation values of IOE (H) with and without considering cell density were 0.05 ± 0.018 and 0.142 ± 0.012, respectively, and show significant differences (P-value = 0.00). Moreover, IOE (H) values showed statistically significant differences from conventional HI values. The results showed that considering cell density in the IOE (H) formula resulted in lower values than conventional HI and IOE(H) without considering cell density.

**Fig. 3:**
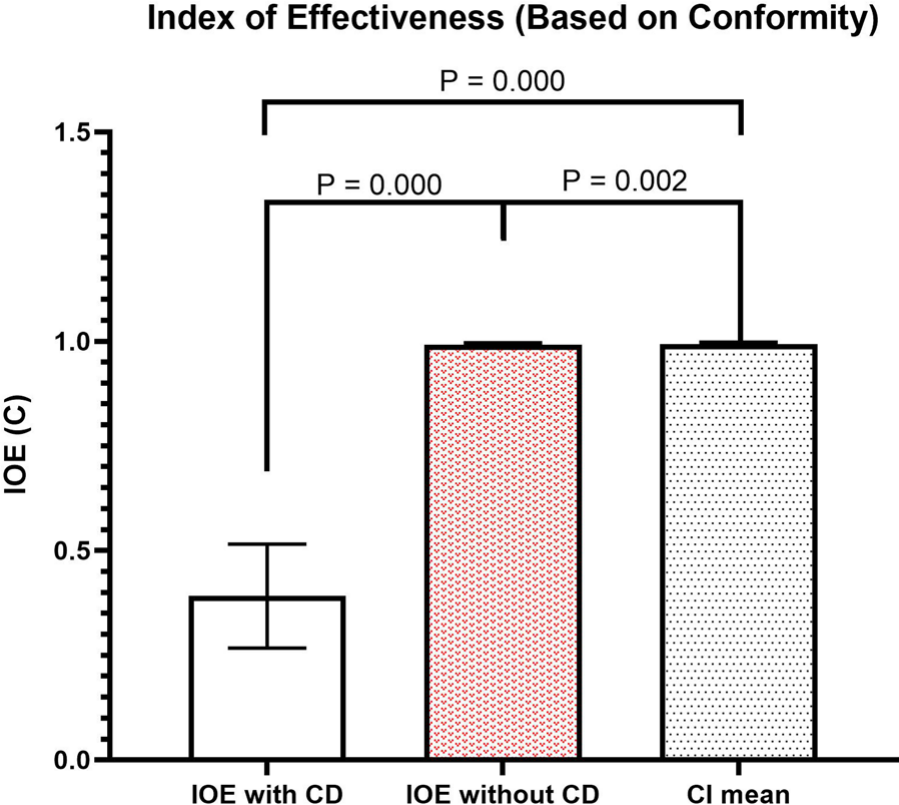
Fig. 2 Mean and standard deviation (error bars) values of the IOE (C) with and without considering cell density coefficients and also the average of conventional CI values. *P-values lower than 0.05 represents the statistically significant differences

Mean and standard deviation values of IOE (C) with and without considering cell density were 0.392 ± 0.124, and 0.993 ± 0.004, respectively (Fig. 3). Furthermore, the mean and standard deviation of conventional CI was 0.994 ± 0.004. The IOE (C) with considering cell density had a significantly lower value compared to conventional CI and IOE (C) without considering cell density (*P*-value ≤ 0.02).

### Correlations between the IOE and tumor control probability values

The mean ± standard deviation values of tumor control probability for CTV_70Gy_ was 94.72 ± 0.65. These values for CTV_66Gy_, and CTV_60Gy_ were 91.58 ± 1.34 and 77.48 ± 3.28 respectively. TCP values greater than 0.7 (70%) have been reported to be "appropriate for total tumor control" in a study by Casares et al. [59].

The Spearman non-parametric correlation was used to investigate the correlation between tumor control probability (TCP), the IOE indices introduced in this study, and conventional HI and CI. The Correlation coefficients between TCP and IOE indices with and without considering cell density were presented in Table 2. Furthermore, the correlation between the TCP and conventional HI and CI values are provided in this table.

**Table 2:** The Correlation coefficients between TCP with IOE indices (with and without considering cell density) and conventional plan evaluation indices (HI and CI)

IOE(H) WITH cellular density	IOE(H) WITHOUT cellular density	IOE(C) WITH cellular density	IOE(C) WITHOUT cellular density	HI (mean)	CI (mean)
-0415	0.152	−0.403	0.624	0.146	0.359

The correlation coefficient between TCP and IOE (HI) with considering cell density showed a moderate and negative correlation. IOE (HI) without cell density coefficient was not correlated with tumor control probability. In addition, the correlation coefficient between TCP and IOE (CI) with considering cell density has a moderate and negative correlation. In contrast, the IOE (CI) without cell density coefficient had a strong and positive correlation with TCP. The correlation of TCP with conventional HI mean and CI means were not significant.

## Discussion

Dose painting radiotherapy is a technique that can produce more targeted dose delivery to tumor-rich regions while saving organs at risk and critical normal tissues. Dose painting planning would be more complicated due to different levels of the prescribed dose levels and harder to evaluate with conventional plan quality indices considering uniform dose prescription. Therefore we tried to introduce new indices for evaluating the conformity and homogeneity based on the tumoral cell density and relative volumes of each lesion in prostate IMRT.

There is a recent study [31] tried to introduce new indices to evaluate the plan quality of inhomogeneous irradiated targets and indicate “achievement” in DP plans based on an introduced IOA (index of achievement) as an alternative to the conventional homogeneity index [60]. Their introduced indices may not necessarily be correlated with the biological effect. The proposed indices in this study can be easily modified to incorporate such an effect. We incorporated the tumor cell density obtained from the DWI and ADC images in the plan evaluation indices, and therefore our introduced indices contain the biological effects. However, the cell density can be obtained from other imaging modalities such as “positron emission tomography” and can be evaluated in future studies.

Applying single or just several indices in plan evaluation is an easy and clinically acceptable method. However, using one or several indexes for plan evaluation can suffer from lacking detailed information. Therefore, it must be mentioned that the introduced indices can not replace the standard tools, such as isodose lines and DVH curves evaluation for treatment plan assessment. Although, they can present additional information. There is an alternative to the standard DVH, delta-volume histogram (ΔVH), which was introduced by Witte et al. [30]. They mainly addressed cumulative ΔVH. But in another study by Park et al. [31], an IOA was introduced, which could be supported by differential ΔVH (dΔVH). It was assumed that each target voxel has an equal amount of impact on the calculation of plan evaluation metrics. However, the impact of each voxel can be different. For example, under-dose regions in a PTV with a higher prescription dose may have clinically higher risks compared to the under-dose regions in a lower dose PTV. This issue can be resolved by adding voxel-specific or region-specific weighting factors to the previous plan evaluation indices. We used cell density obtained from the ADC map for different PTVs as region-specific weighting factors. We observed that the IOE values based on cellular densities could be completely different when applying the weighting factor. If the biological importance of hotness and coldness becomes much more apparent, a more accurate weighting factor system can be found in the future.

We evaluate the relationship of our proposed indices with TCP. Analytical radiobiological parameters (such as TCP and normal tissue complication probability (NTCP)) have been widely used for evaluating the quality of treatment plans [61–63]. In particular, several studies proposed TCP models for inhomogeneously irradiated tumors or planning target volumes [18, 64]. Our results showed that IOE(H) with considering cell density and IOE(C) without considering cell density had a stronger relationship with TCP. Therefore it may be concluded that considering cell density values in calculating IOE(C) was not an appropriate idea. However, cell densities must be included in the IOE(H) calculation formula.

The cell density values used in our proposed formula of IOE indices were calculated based on the ADC map. Furthermore, the dose escalation was also based on the regions extracted based on these ADC images. A number of previous studies have advocated the strategy of dose escalation to the imaging-defined targets and dose de-escalation to the rest of the prostate. High dose areas in DP plans can increase the delivery uncertainty due to the limited capability of the treatment planning optimization algorithm to deliver high doses to small and isolated areas. Therefore, these high-dose areas must be defined accurately, and the method of determining these areas must have high repeatability. Comparable findings on the repeatability of ADC features in MRI prostate imaging are reported in the literature. Toivonen et al. [35] reported an ICC of 0.89 for ADC intensity in prostate cancer using MRI, although performed on an ROI basis. Koh et al. also reported high repeatability for ADC measurements in a two-center phase I clinical trial [65].

Van Lin et al. [66] performed a dose panting planning study on five patients with standard whole-prostate RT conventional plan to 78 Gy and a plan with DIL dose escalation to 90 Gy based on dynamic contrast-enhanced and 1H-spectroscopic MRI, and the remainder of the prostate dose de-escalation to 70 Gy. They reported that both plans had similar TCPs; however, the dose painting had lower NTCPs. In another study by Seppala et al. [21], a planning study of 12 patients was performed with DILs defined based on ^11^C acetate PET scans. Six different dose escalation plans were performed and compared for each patient, including a whole-prostate RT plan to 77.9 Gy, and DIL dose escalations to 77.9 Gy, 81 Gy, 84 Gy, 87 Gy and 90 Gy, with remaining prostate dose de-escalations to 72 Gy. They reported that the dose painting plans had higher TCP values compared with the standard whole-prostate plan and that the highest probability of tumor control without complication was related to a plan with an average dose of 82.1 Gy to the DIL. In a study by Chang et al.[23] the technical feasibility of IMRT dose painting using ^11^C-choline PET scans were evaluated in eight patients with localized prostate cancer. Two DILs were defined including 60% and 70% of the maximum standardized uptake values (SUV_60%_ and SUV_70%_). Three IMRT plans were designed including: PLAN_78_ (whole-prostate irradiation with 78 Gy); PLAN_78-90_ (whole-prostate RT to 78 Gy, a boost to the SUV_60%_ and SUV_70%_ to 84 Gy, and 90 Gy, respectively); and PLAN_72-90_ (whole-prostate RT to 72 Gy, a boost to the SUV_60%_ and SUV_70%_ to 84 Gy, and 90 Gy, respectively). TCP based on PET scan-defined volumes (TCP_PET_) and on prostatectomy-defined volumes (TCP_path_), and rectal NTCP were compared between the plans. They reported that both dose painting plans (PLAN_78-90_ and PLAN_72-90_) had significantly higher TCP_PET_ and TCP_path_ values than conventional IMRT plan (PLAN_78_), without significant differences in TCP_PET_ or TCP_path_ between dose painting plans. Furthermore, There were no significant differences in rectal NTCPs between the 3 plans.

We used rigid image registration for fusing CT and MRI images. Deformable registration can also be used for this purpose. Rigid registration is very effective in cases when no anatomic change is expected [67]. In our study, patients underwent MR imaging after CT imaging in one day with a maximum delay of one hour. Their positioning was similar in both imaging and therefore, we don’t expect significant anatomical changes between the imaging techniques. If there is a big time gap between CT and MR imaging, patients might experience anatomical changes due to tumor shrinkage/growth, weight loss, or physiological organ shape variations. In these cases deformable registration can manage the distortion between two image sets and provide superior results [68–70]. In comparison to rigid registration, deformable registration has a significantly greater degrees of freedom [67], and can deform the image and structures with different algorithms (such as intensity-based approaches, landmark-based thin-plate spline, or biophysical and finite element modelling-based registration) (67).

This study has some limitations, and several factors must be addressed before clinically adopting this strategy. First, deformable registration might be superior in cases with the significant time intervals between CT and MR imaging because this could deal with changes in the prostate shape and discrepancies in the prostate size between imaging modalities more adequately than was possible using rigid registration. Second, the proposed plan evaluation indices in this study, including IOE(H) and IOE(c) with and without considering cell densities, must be assessed for a bigger group of patients and also in other cancer sites.

## Conclusions

New IOE dose painting plan evaluation indices proposed in this study have simple calculation methods and incorporate cellular density as an index of lesion importance obtained from MRI ADC images for prostate cancer IMRT. These indices can be used for evaluating prostate IMRT dose painting plans. Cell densities must be considered in the IOE(H) (calculation formula, and it's more appropriate to calculate IOE(C) without considering cell density.

## Supplementary Information

**Additional file 1.** An in-house MATLAB program developed to automatically identify lesion regions on ADC images based on ADC values.

## Data Availability

All data obtained during the current study are available from the corresponding author on reasonable request.

## Abbreviations

IMRT: Intensity modulated radiotherapy
VMAT: Volumetric modulated arc therapy
IMAT: Intensity modulated arc therapy
ADC: Apparent diffusion coefficient
GTV: Gross tumor volume
CTV: Clinical tumor volume
PTV: Planning tumor volume
HI: Homogeneity index
CI: Conformity index
IOE: Index of effectiveness
DVH: Dose volume histogram
MRI: Magnetic resonance imaging
CT: Computed tomography
