# Supplementary material for: Introducing new plan evaluation indices for prostate dose painting IMRT plans based on apparent diffusion coefficient images
Source: Radiat Oncol. 2022 Nov 23;17:193. doi: 10.1186/s13014-022-02163-7 (PMC9685857; doi:10.1186/s13014-022-02163-7)
Supplement: Supplementary file 1 — Additional file 1. An in-house MATLAB program developed to automatically identify lesion regions on ADC images based on ADC values. [file 13014_2022_2163_MOESM1_ESM.docx]

## Supplementary Materials

**MATLAB program code**

The in-house MATLAB code developed to automatically identify lesion regions on MRI images based on ADC values is presented as follows:

%%%%%%%%%%% This program is Written by "Saman Moradi" under the supervision of "Dr. Bijan Hashemi" %%%%%%%%%%%

%%%%%%%%%%% Using this program is free subject to citing this article and taking the permission from one of the persons mentioned above, available at:

%%%%%%%%%%% bhashemi@modares.ac.ir%%%%%%%%%%%%%%

clc

clear all;

close all;

%% Read Input Image

InputImage = dicomread('D:\Patients\Series_6_ep2d_diff_b0-250-500-1000\15.dcm');

%% Display the Image

imshow(InputImage,[]);

str = 'Draw initial contour location in Image. Double-click to confirm and proceed.';

title(str,'Color','r','FontSize',10);

%% Get Inputs from Mouse, draw contour in Image:

mask = drawfreehand;

Col = mask.Position(:,1);

Row = mask.Position(:,2);

D =Col;

r =Row;

%% Select polygonal region of interest

BinaryMask = roipoly(InputImage,D,r);

%% Create Buffer for ROI

ROI = zeros(128,128);

%% Create Buffer for NONROI

NONROI = zeros(128,128);

for i = 1:128

for j = 1:128

if BinaryMask(i,j)==1

ROI(i,j) = InputImage(i,j);

else

NONROI(i,j) = InputImage(i,j);

end

end

end

%% Display ROI and Non ROI

figure;

subplot(1,2,1);

imshow(ROI,[]);title('ROI');

subplot(1,2,2);

imshow(NONROI,[]);title('NON ROI');

%% Display segmented image

figure, imshow(ROI,[])

title('Segmented Image');

%% Overlay contours on the image

figure;

subplot(1,2,1);

imshow (NONROI,[0,3000]);

str = 'Two types of lisons:';

title(str,'Color','r','FontSize',10);

hold on

CONTOURED_TOTAL = contour (ROI,1:1:5000,'w');

CONTOURED_0 = contour (ROI,1501:1:5000,'m');

CONTOURED_1 = contour (ROI,0:1:750,'r');

CONTOURED_2 = contour(ROI,751:1:1500,'g');

subplot(1,2,2);

imshow (NONROI,[0,3000]);

str = 'Total lison side:';

title(str,'Color','r','FontSize',10);

hold on

CONTOURED_3 = contour (ROI,0:1:1300,'c');

CONTOURED_4 = contour (ROI,1301:1:5000,'color','[ 0.9100 0.4100 0.1700]');

%% CALCULATING MEAN OF A.D.C. NUMBERS FROM "0" TO "750" FROM ROI OF PATIENTS IMAGES.

A = CONTOURED_1(1,:);

index_A = 1;

result_A = [];

counter_A = 0;

for i = 1 : length(A)

if (i== length(A))

result_A = [result_A; { counter_A } ];

break

end

number = CONTOURED_1(2,i);

integ = floor(number);

fract = number-integ;

if ((fract == 0.0000))

s = single(CONTOURED_1(2,i));

int = uint16(s);

TF = isinteger(int);

if (A(1,i) == index_A && TF == true)

result_A = [result_A; { counter_A } ];

index_A = index_A + 1;

counter_A = 0;

continue

end

end

counter_A = counter_A + 1 ;

end

result_A = result_A(2:end, 1);

result_mat_A = cell2mat(result_A);

for k = 1 : 750

num_A(k,1) = k;

end

multiplication_A = num_A .* result_mat_A;

sum1_A = sum(multiplication_A);

sum2_A = sum(result_mat_A);

RESULT_FINAL_A = sum1_A/sum2_A

%% CALCULATING MEAN OF A.D.C. NUMBERS FROM "751" TO "1500" FROM ROI OF PATIENTS IMAGES.

B = CONTOURED_2(1,:);

index_B = 751;

result_B = [];

counter_B = 0;

for i = 1 : length(B)

if (i == length(B))

result_B = [result_B; { counter_B } ];

break

end

number = CONTOURED_2(2,i);

integ = floor(number);

fract = number-integ;

if (fract == 0.0000)

s = single(CONTOURED_2(2,i));

int = uint16(s);

TF = isinteger(int);

if (B(1,i) == index_B && TF == true)

result_B = [result_B; { counter_B } ];

index_B = index_B + 1;

counter_B = 0;

continue

end

end

counter_B = counter_B + 1 ;

end

result_B = result_B(2:end, 1);

result_mat_B = cell2mat(result_B);

result_mat_B_shifted = zeros(1500, 1);

result_mat_B_shifted(751:(750+(numel(result_mat_B))),1) = result_mat_B(1:end,1);

for k = 1 : 1500

num_B(k,1) = k;

end

multiplication_B = num_B .* result_mat_B_shifted;

sum1_B = sum(multiplication_B);

sum2_B = sum(result_mat_B_shifted);

RESULT_FINAL_B = sum1_B/sum2_B

%% CALCULATING MEAN OF A.D.C. NUMBERS FROM "1501" TO "5000" FROM ROI OF PATIENTS IMAGES.

C = CONTOURED_0(1,:);

index_C = 1501;

% result_C = [];

result_C = cell(1501, 1);

result_C(1:1500, 1) = {0};

counter_C = 0;

for i= 1 : length(C)

if (i == length(C))

result_C = [result_C; { counter_C } ];

break

end

number = CONTOURED_0(2,i);

integ = floor(number);

fract = number-integ;

if ((fract == 0.0000))

s = single(CONTOURED_0(2,i));

int = uint16(s);

TF = isinteger(int);

if ( (C(1,i)== index_C) && (TF == true) )

result_C = [result_C; { counter_C } ];

index_C = index_C + 1;

counter_C = 0;

continue

end

end

counter_C = counter_C + 1 ;

end

result_C = result_C(2:end, 1);

result_mat_C = cell2mat(result_C);

result_mat_C_shifted = zeros(5000,1);

result_mat_C_shifted(1:((numel(result_mat_C))),1) = result_mat_C(1:end,1);

for k = 1:5000

num_C(k,1) = k;

end

multiplication_C = num_C .* result_mat_C_shifted;

sum1_C = sum(multiplication_C);

sum2_C = sum(result_mat_C_shifted);

RESULT_FINAL_C = sum1_C/sum2_C

%% CALCULATING MEAN OF A.D.C. NUMBERS FROM "0" TO "5000" FROM ROI OF PATIENTS IMAGES.

TOTAL = CONTOURED_TOTAL(1,:);

index_TOTAL = 1;

result_TOTAL = [];

counter_TOTAL = 0;

for i= 1 : length(TOTAL)

number = CONTOURED_TOTAL(2,i);

integ = floor(number);

fract = number-integ;

if ((fract == 0.0000))

s = single(CONTOURED_TOTAL(2,i));

int = uint16(s);

TF = isinteger(int);

if ( (i== length(TOTAL)) && (fract == true))

result_TOTAL = [result_TOTAL; { counter_TOTAL } ];

break

end

if TOTAL(1,i)== index_TOTAL

result_TOTAL = [result_TOTAL; { counter_TOTAL } ];

index_TOTAL = index_TOTAL + 1;

counter_TOTAL = 0;

continue

end

end

counter_TOTAL = counter_TOTAL + 1 ;

end

result_TOTAL = result_TOTAL(2:end, 1);

result_mat_TOTAL = cell2mat(result_TOTAL);

result_mat_TOTAL_shifted = zeros( numel(result_mat_TOTAL), 1);

result_mat_TOTAL_shifted((1:(numel(result_mat_TOTAL))), 1) = result_mat_TOTAL(1:end, 1);

for k = 1 : (numel(result_mat_TOTAL))

num_TOTAL(k,1) = k;

end

multiplication_TOTAL = num_TOTAL .* result_mat_TOTAL_shifted;

sum1_TOTAL = sum(multiplication_TOTAL);

sum2_TOTAL = sum(result_mat_TOTAL_shifted);

RESULT_FINAL_TOTAL = sum1_TOTAL/sum2_TOTAL

%% CALCULATING MEAN OF A.D.C. NUMBERS FROM "0" TO "1300" FROM ROI OF PATIENTS IMAGES.

D = CONTOURED_3(1,:);

index_D = 1;

result_D = [];

counter_D = 0;

for i= 1 : length(D)

if (i== length(D))

result_D = [result_D; { counter_D } ];

break

end

number = CONTOURED_3(2,i);

integ = floor(number);

fract = number-integ;

if ((fract == 0.0000))

s = single(CONTOURED_3(2,i));

int = uint16(s);

TF = isinteger(int);

if ( (D(1,i)== index_D) && ( TF == true) )

result_D = [result_D; { counter_D } ];

index_D = index_D + 1;

counter_D = 0;

continue

end

end

counter_D = counter_D + 1 ;

end

result_D = result_D(2:end, 1);

result_mat_D = cell2mat(result_D);

result_mat_D_shifted = zeros(1300, 1);

result_mat_D_shifted((1:(numel(result_mat_D))), 1) = result_mat_D(1:end,1);

for k = 1 : 1300

num_D(k,1) = k;

end

multiplication_D = num_D .* result_mat_D_shifted;

sum1_D = sum(multiplication_D);

sum2_D = sum(result_mat_D_shifted);

RESULT_FINAL_D = sum1_D/sum2_D

%% CALCULATING MEAN OF A.D.C. NUMBERS FROM "1301" TO "5000" FROM ROI OF PATIENTS IMAGES.

E = CONTOURED_4(1,:);

index_E = 1301;

% result_E = [];

result_E = cell(1301, 1);

result_E(1:1300, 1) = {0};

counter_E = 0;

for i= 1 : length(E)

if (i == length(E))

result_E = [result_E; { counter_E } ];

break

end

number = CONTOURED_4(2,i);

integ = floor(number);

fract = number-integ;

if ((fract == 0.0000))

s = single(CONTOURED_4(2,i));

int = uint16(s);

TF = isinteger(int);

if ( (E(1,i)== index_E) && TF == true)

result_E = [result_E; { counter_E } ];

index_E = index_E + 1;

counter_E = 0;

continue

end

end

counter_E = counter_E + 1 ;

end

result_E = result_E(2:end, 1);

result_mat_E = cell2mat(result_E);

result_mat_E_shifted = zeros(5000, 1);

result_mat_E_shifted(1:((numel(result_mat_E))),1) = result_mat_E(1:end,1);

for k = 1 : 5000

num_E(k,1) = k;

end

multiplication_E = num_E .* result_mat_E_shifted;

sum1_E = sum(multiplication_E);

sum2_E = sum(result_mat_E_shifted);

RESULT_FINAL_E = sum1_E/sum2_E

%%%%%%%%%%%%%%%%%%%%%%%%%% End %%%%%%%%%%%%%%%%%
